# Structural and magnetic properties of microwave-synthesized reduced graphene oxide/VO_2_/Fe_2_O_3_ nanocomposite

**DOI:** 10.3762/bjnano.16.70

**Published:** 2025-06-20

**Authors:** Sumanta Sahoo, Ankur Sood, Sung Soo Han

**Affiliations:** 1 School of Chemical Engineering, Yeungnam University, 280 Daehak-ro, Gyeongsan 38541, South Korea

**Keywords:** Fe_2_O_3_, magnetism, microwave irradiation, reduced graphene oxide, VO_2_

## Abstract

Reduced graphene oxide (rGO)-assisted microwave (MW) synthesis of metal-oxide-based binary and ternary nanocomposites has recently gained considerable research attention. In this context, the current work demonstrates a facile rGO-supported solid-state MW synthetic route for fabricating a ternary nanocomposite of VO_2_, Fe_2_O_3_, and rGO. Here, the MW irradiation for 90 s was found to be suitable for the reduction and exfoliation of graphite oxide to form rGO, the reduction of V_2_O_5_ to form VO_2,_ and the formation of Fe_2_O_3_ from ferrocene. X-ray diffraction and X-ray photoelectron spectroscopy analyses confirm the formation of distinct metal oxides in the presence of rGO. Furthermore, the morphological analysis reveals the deposition of Fe_2_O_3_ nanoparticles and VO_2_ nanorods on the 2D rGO surface. Notably, the ternary composite displayed good magnetic properties for its potential biomedical applications. Overall, this work explores an efficient and cost-effective synthetic approach for developing graphene-based magnetic nanocomposites.

## Introduction

Graphene-based materials have been significantly explored in various fields of materials science due to their unique physical and chemical characteristics [1–4]. The special arrangement of carbon materials in a honeycomb fashion enabled this special class of materials to exhibit desirable characteristics, such as enhanced electrical conductivity, higher mechanical strength, elevated surface area, and high thermal and chemical stability. Owing to such improved characteristics, graphene materials, including their derivatives, are broadly explored for miscellaneous applications, such as energy storage/conversion, EMI shielding, biosensing, optoelectronics, robotics, flexible electronics, paint industries, textile industries, biomedical devices [5–7]. To be specific, the innovation of graphene unlocked a new era in the field of materials science.

The synthetic approaches of graphene materials including graphene quantum dots, graphene oxide, and reduced graphene oxide (rGO) can be categorized into two classes: top-down and bottom-up processes. The top-down approaches are found to be comparatively simpler and cost-effective compared to the bottom-up processes. Among the top-down approaches, the microwave (MW)-assisted exfoliation process of synthesizing graphene materials and related nanocomposites (NCs) has gained noteworthy research attention in recent times [8–9]. For synthesizing binary and ternary NCs of graphene materials, several conventional approaches, such as sol–gel, hydrothermal/solvothermal, calcination/thermal annealing, chemical vapor deposition, liquid-phase exfoliation, and freeze-drying have been reported. However, the MW-assisted synthetic approaches are found to be superior to these approaches due to lesser time consumption and the lack of sophisticated instrumentation. In this aspect, Kumar et al. demonstrated a facile MW-assisted synthetic route for the perforation and decoration of Pd nanoparticles (NPs) on rGO sheets [10]. The resultant NC displayed its potential for supercapacitor applications. In another work, Mn_3_O_4_ nanograins-intercalated rGO NC was synthesized through the MW-assisted hydrothermal approach, which showed superior oxygen reduction reaction (ORR) activity [11]. Aside from the oxides, mixed metal sulfides are likewise reported to be combined with graphene by applying MW irradiation for certain time intervals. In this context, Zhang et al. reported the synthesis of Ni-Co sulfide/graphene NC through MW irradiation at a power of 1000 W for 60 s [12]. As a supercapacitor (SC) electrode, the MW-synthesized NC displayed a specific capacitance of 710 F/g.

The MW-assisted approaches have been further explored for the synthesis of binary NCs based on rGO and iron oxides. In one of our earlier reports, such an MW irradiation-based approach was adopted to synthesize Fe_2_O_3_/rGO NC, using hemin as the precursor. Owing to its 3D network structure, the NC displayed good electrochemical performance as the SC electrode [13]. In another work, Kumar et al. reported the MW-assisted synthesis of Fe_3_O_4_/rGO NC using FeCl_3_ aqueous salt as the precursor [14]. Ferrocene, an Fe-based organometallic compound, was also utilized as the precursor for developing NCs of iron oxide and graphene through the MW route. For example, Kumar et al. demonstrated the MW-assisted rapid synthesis of a ternary NC based on rGO, carbon nanotubes, and Fe_3_O_4_ NPs, using ferrocene as the Fe-containing precursor [15]. The NC exhibited its potential to be used for EMI shielding applications.

Inspired by previous research findings, the current article aims to produce a ternary NC based on rGO, VO_2_, and Fe_2_O_3_ using ultrafast MW irradiation. The applied MW irradiation of only 90 s was found to be beneficial for reducing as well as exfoliating graphite oxide to form rGO. At the same time, the approach was also able to convert V_2_O_5_ to form VO_2_ and synthesize Fe_2_O_3_ from ferrocene. The structure and properties of the NC were examined through various characterization techniques. Lastly, the magnetic properties of Fe-containing ternary NCs were also evaluated for their possible biomedical applications.

## Experimental

### Materials

Vanadium (V) oxide (V_2_O_5_) powder and ferrocene were purchased from Alfa Aesar. The other chemicals used for the synthesis of graphite oxide, such as conc. sulfuric acid (H_2_SO_4_), hydrogen peroxide (H_2_O_2_), conc. hydrochloric acid (HCl), potassium chlorate (KClO_3_), conc. nitric acid (HNO_3_), and ethanol were obtained from Duksan Pure Chemicals Co. Ltd. Graphite powder was supplied by Sigma-Aldrich.

### Instrumentation and characterization techniques

The “PANalytical, X’Pert-PRO MPD” instrument (Cu Kα line; λ = 1.5406 Å) was utilized to carry out the XRD analyses of rGO and the NCs. The Raman spectra of rGO and the related NCs were recorded through the “XploRA plus HORIBA” instrument with a laser excitation of 532 nm. Additionally, the surface analysis was performed using X-ray photoelectron spectroscopy measurements (XPS, Thermofisher Scientific) functioning at 12 kV and 6.50 mA using an Al Kα. The morphologies and elemental analyses of rGO and the NCs were analyzed through scanning electron microscopy (SEM, Hitachi, S-4800). The structural analysis of these fabricated NCs was examined using high-resolution transmission electron microscopy (HRTEM, FEI Tecnai G2 F20). Furthermore, the magnetic properties of the NCs were evaluated using a vibrating-sample magnetometer (VSM, LakeShore (8604)).

### Microwave synthesis of reduced graphene oxide

Following a previous report, graphite powder was initially oxidized to form graphite oxide to synthesize rGO through MW irradiation [16]. In the next step, 200 mg of the graphite oxide was MW irradiated at 700 W for 90 s in a MW oven. It is important to note that the MW process happened in the solid phase. The reduction and exfoliation of graphite oxide to form the rGO occurred through the removal of oxygen functionalities in the gaseous form. It is interesting to note that the obtained rGO material is found to be much lighter than the graphite oxide precursor.

### Microwave synthesis of rGO/VO_2_ nanocomposite

The solid-state MW irradiation process was followed to synthesize the GV NC. In a typical process, initially, the graphite oxide (100 mg) was mixed with V_2_O_5_ powder (100 mg) in a mortar pestle. In the next step, the mixed powder was MW irradiated at a power of 700 W for 90 s to obtain the GV NC.

### Microwave synthesis of rGO/VO_2_/Fe_2_O_3_ nanocomposite

The ternary NC was synthesized following a similar solid-state MW irradiation process. It is important to note that ferrocene was used as the precursor for the iron oxide. In a typical process, graphite oxide (100 mg), V_2_O_5_ powder (50 mg), and ferrocene (50 mg) were thoroughly mixed in a mortar pestle. Finally, the mixed powder was MW irradiated at a power of 700 W for 90 s to synthesize the ternary GVF.

## Results and Discussion

**Figure 1:**
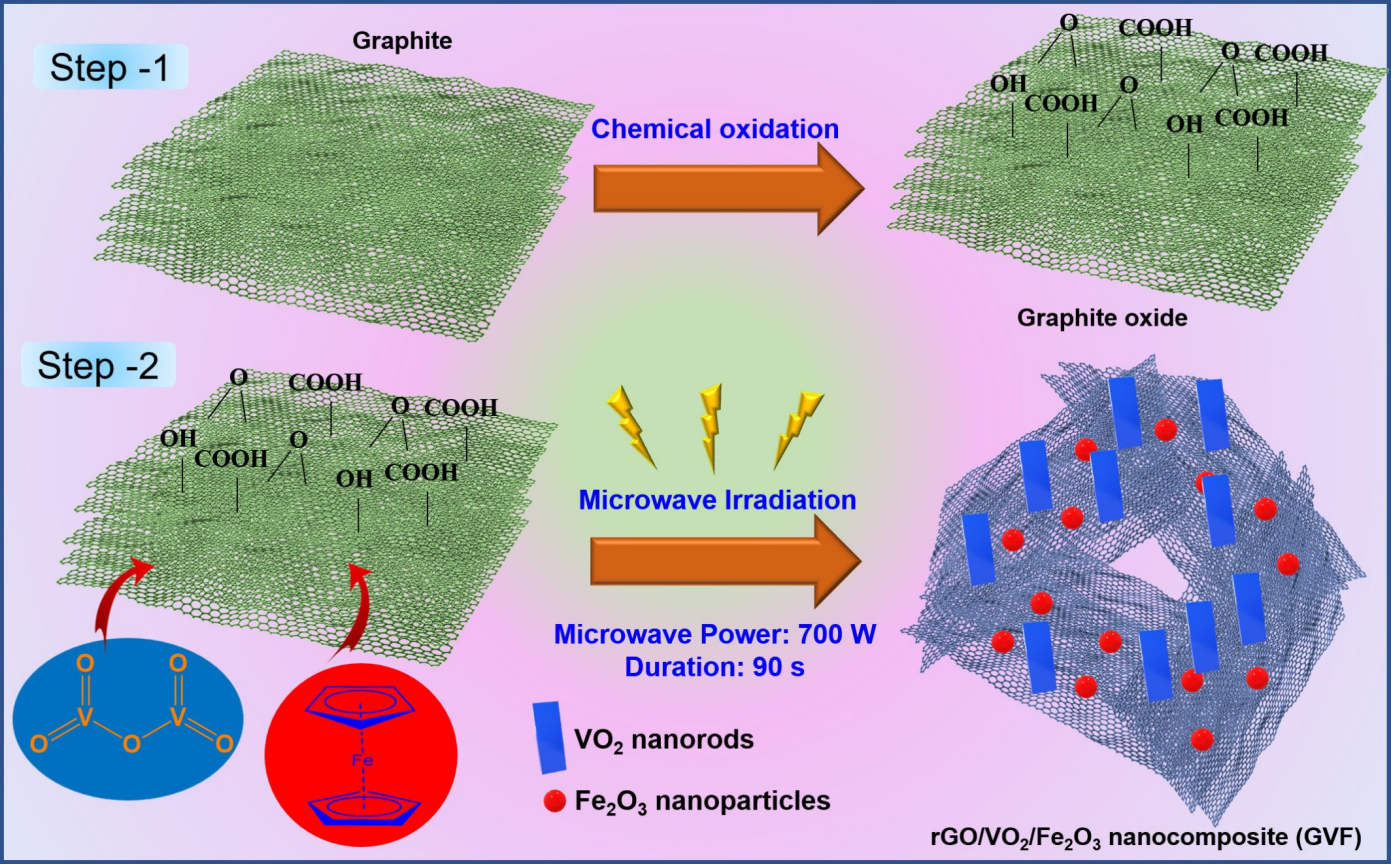
Schematics of the synthetic approach of GVF NC.

The NC based on rGO, VO_2_, and Fe_2_O_3_ was synthesized through a cost-effective, ultrafast MW route. As shown in Figure 1, the graphite powder was initially oxidized through a chemical synthetic route to form graphite oxide. In the next step, the MW irradiation of constant power for a fixed time duration was applied to form the NC. It is evident that the NCs of Fe_2_O_3_ and graphene materials are usually synthesized through hydrothermal/solvothermal processes.

In this aspect, a comparative study of a few ternary NCs based on Fe_2_O_3_ and graphene materials is shown in Table 1. Most of the reported synthetic processes are composed of multiple steps, are time-consuming, and utilize higher temperatures. Compared to these approaches, the adopted synthetic route has the advantages of less time and energy consumption. Moreover, the solid-state MW irradiation was conducted inside the MW oven at room temperature. Furthermore, most of the reported works are based on synthesizing Fe_2_O_3_ from Fe-based aqueous salt in liquid phases. In contrast, the current work demonstrates the formation of Fe_2_O_3_ from a Fe-based organometallic compound, ferrocene.

**Table 1:** Comparative study of the reaction conditions of a few ternary NCs based on Fe_2_O_3_ and rGO.

Nanocomposite (NC)	Fe Precursor	Synthetic approach	Conversion time for iron oxide	Conversion temperature for iron oxide	Ref.

graphene/Fe_2_O_3_/polyaniline	FeCl_2_·4H_2_O	hydrothermal + in situ polymerization	10 h	180 °C	[17]
rGO/Fe_2_O_3_/SnO_2_	FeCl_3_·6H_2_O	hydrothermal + thermal annealing	4 h (hydrothermal) 1 h (thermal annealing)	120 °C 400 °C	[18]
Fe_2_O_3_/NiO/rGO	ferric nitrate	hydrothermal + MW heating	5 h (hydrothermal) 10 min (MW)	180 °C 700 °C	[19]
C_3_N_4_/Fe_2_O_3_/V_2_O_5_	FeCl_3_·6H_2_O	stirring + hydrothermal + thermal annealing	5 h (stirring) 12 h (hydrothermal) 2 h (annealing)	60 °C (stirring) 160 °C (hydrothermal) 500 °C (annealing)	[20]
C_3_N_4_/Fe_2_O_3_/graphene aerogel	FeCl_3_·6H_2_O	stirring + hydrothermal + freeze drying	5 h (stirring) 12 h (hydrothermal)	80 °C (stirring) 180 °C (hydrothermal)	[21]
rGO/Fe_2_O_3_/polyindole	Fe(NO_3_)_3_·9H_2_O	hydrothermal + MW irradiation	24 h (hydrothermal) 3 min (MW irradiation)	180 °C (hydrothermal)	[22]
C_3_N_4_/Ti_3_C_2_/Fe_2_O_3_	FeCl_3_·6H_2_O	solvothermal	12 h	180 °C	[23]
C_3_N_4_/Fe_2_O_3_/CdS	FeCl_3_·6H_2_O	calcination	4 h	400 °C	[24]
rGO/VO_2_/Fe_2_O_3_	ferrocene	MW irradiation	90 s	room temperature	this work

**Figure 2:**
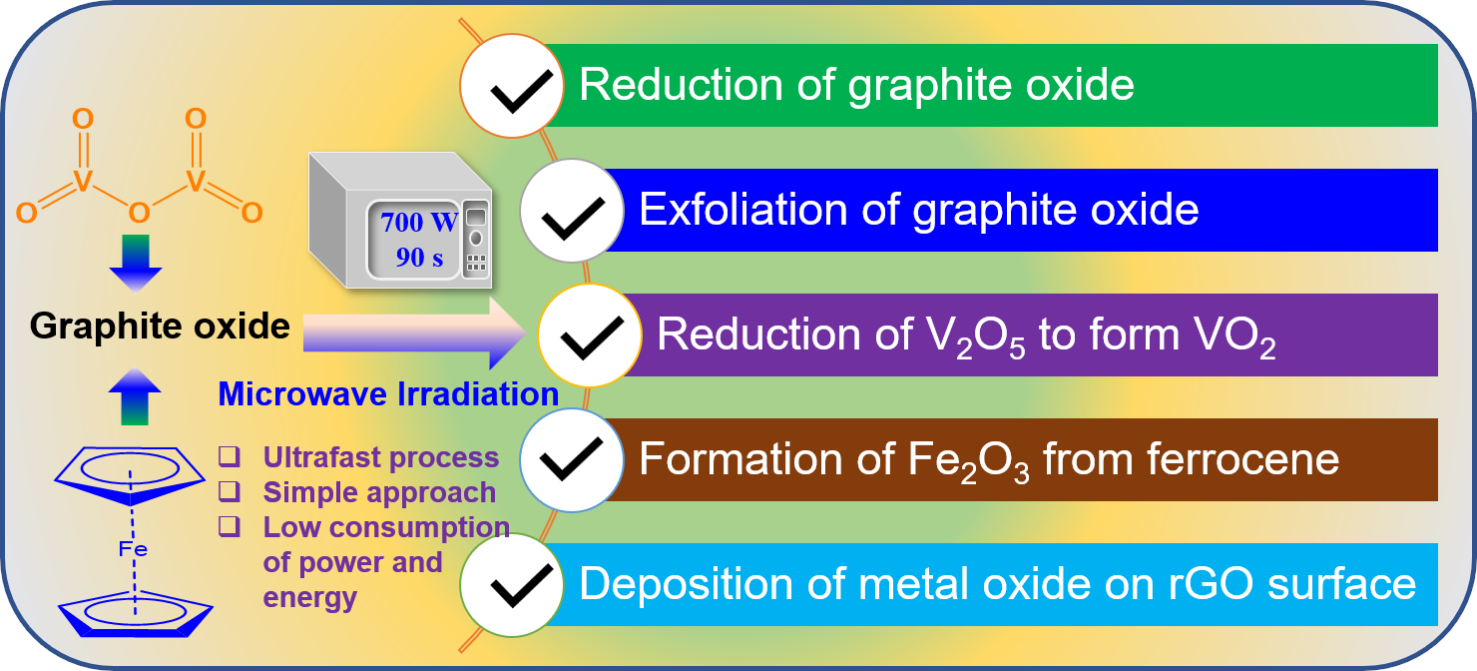
Schematics representing the effects of MW irradiation on the mixed powder of graphite oxide, V_2_O_5_, and ferrocene.

Herein, the graphite oxide was utilized as an MW susceptor, which deliberately absorbed the MW irradiation and generated heat. The generation of heat was caused by the interaction of oxygen functionalities with the MW irradiation. The produced heat was capable enough for the conversion of V_2_O_5_ and ferrocene to form the corresponding oxides, which eventually dispersed on the surface of the graphene sheets. In the meantime, the graphite oxide was reduced and exfoliated to form rGO. The oxygen functionalities present in the graphite oxide were partially removed from the reaction system in the form of gases (e.g., CO_2_, CO) [25]. On the other hand, the remaining oxygen-containing functional groups on the graphene surface acted as a good support for the attachment of metal oxide NPs on the graphene surface. It is important to note that the resultant rGO was found to be much lighter than the graphite oxide precursor, which can be ascribed to significant exfoliation of the graphite oxide to form the rGO. Figure 2 schematically summarizes the effects of MW irradiation on the mixed powder of graphite oxide and metal oxide precursors.

The short-life MW irradiation was also successful in converting V_2_O_5_ to VO_2_. This type of phase change of this metal oxide is generally implemented through a few approaches, including annealing, sol–gel process, hydrothermal process, vapor transport method. However, such approaches are time consuming [26–28]. In this context, the ultrafast reduction of V_2_O_5_ to VO_2_ through the MW route is highly beneficial. On the other hand, the formation and dispersion of Fe_2_O_3_ NPs on the rGO surface through the decomposition of ferrocene followed a similar mechanism, as discussed in a previous report [29]. Upon MW irradiation, the Fe molecules were oxidized to form Fe_2_O_3_ and deposited on rGO surfaces through substantial interactions.

**Figure 3:**
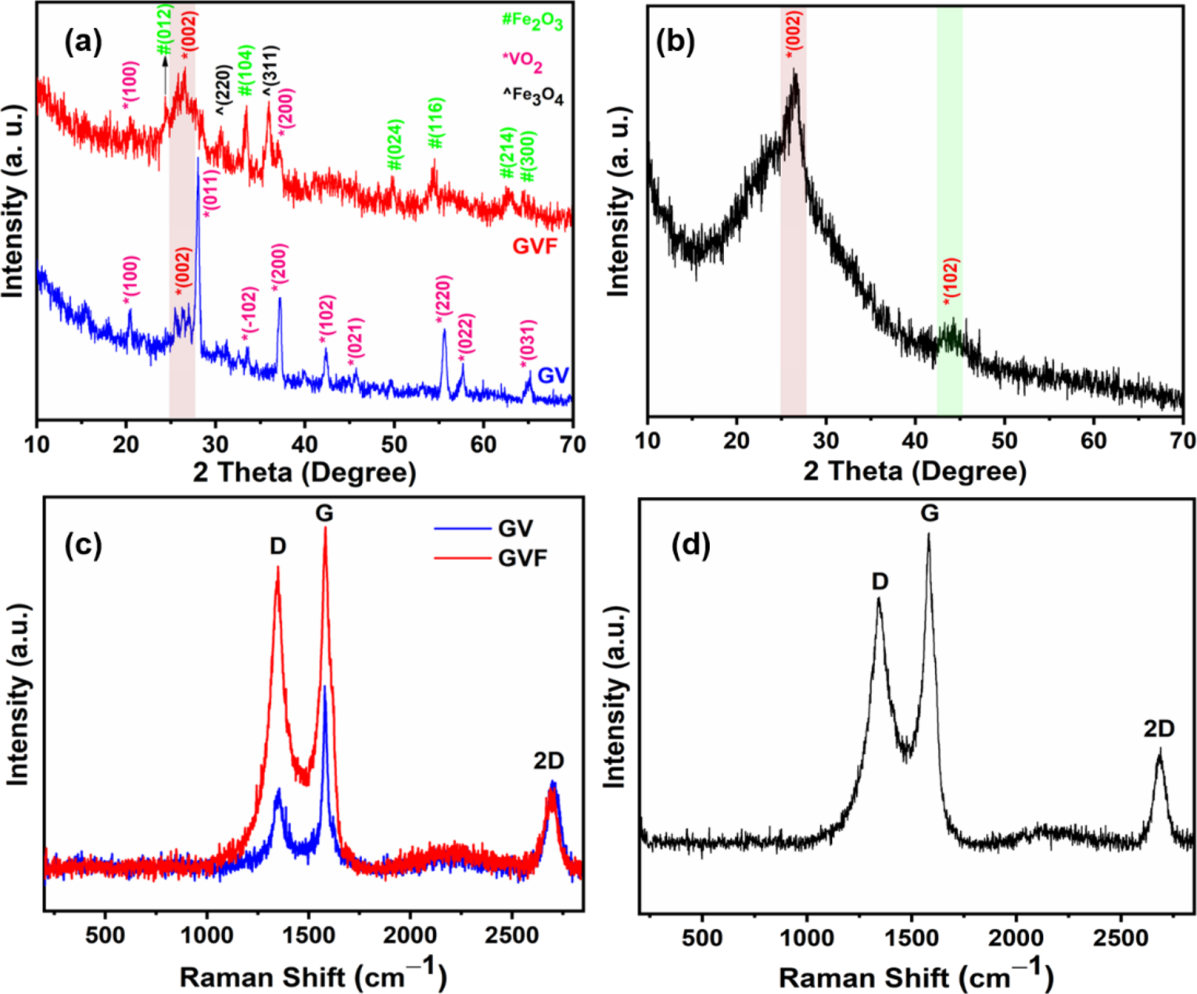
XRD patterns of (a) GV and GVF, (b) rGO; Raman spectra of (c) GV and GVF, and (d) rGO.

To confirm the formation of the ternary NC, the XRD analysis of the GVF was performed. As shown in Figure 3a, the characteristic peaks of GVF corresponding to the α phase of Fe_2_O_3_ and VO_2,_ along with the signature peak of rGO were observed. To be specific, the peaks at the diffraction angles of 24.4°, 33.5°, 49.8°, 54.4°, 62.8°, and 64.4° indicate the (012), (104), (024), (116), (214), and (300) planes of Fe_2_O_3_, respectively (JCPDS Card No. 79-0007) [30]. On the other hand, the high-intensity peak at the diffraction angle of 26.5° represents the characteristic (002) plane of rGO. Notably, other high-intensity peaks at 2θ = 30.6° and 36° could be assigned to the (220) and (311) planes of Fe_3_O_4_, respectively, according to the JCPDS card no. 65-3107 [31]. The presence of such peaks could be accounted for the partial formation of the Fe_3_O_4_ phase of iron oxide while oxidizing ferrocene alongside Fe_3_O_4_, which is formed as a major iron oxide component during the MW irradiation process. Nevertheless, the XRD pattern of the GV demonstrates the characteristic peaks of VO_2_, according to the JCPDS card no. 01-072-0514 [32]. Specifically, the peaks at the diffraction angle of 20.4°, 28°, 33.5°, 37.2°, 42.3°, 45.8°, 55.5°, 57.6°, and 65.2° can be ascribed to the characteristic (100), (011), (−102), (200), (210), (021), (220), (022), and (031) planes of VO_2_. The characteristic peak of the (002) plane of rGO is also exhibited in the diffraction pattern of GV. It is important to note that the diffraction pattern of GVF also displays a few characteristic peaks of VO_2_ with smaller intensity values, indicating its minimal presence in the formed GVF. Additionally, slight shifting of the peak position of individual components in the NCs is an indication of interaction between them. For a comparative study, the XRD pattern of MW-synthesized rGO is shown in Figure 3b. As shown, the high-intensity peak at the diffraction angle of 26.5° corresponds to the (002) plane, and the low-intensity peak at the diffraction angle of 44.2° represents the (102) plane of graphene [33–35]. The presence of rGO in the GV and GVF was further confirmed by the Raman spectra (Figure 3c). As shown, the peaks at ≈1350 and ≈1580 cm^−1^ correspond to the characteristic D and G bands of graphene. Interestingly, minor changes in the peak positions of these two characteristic peaks indicate the alteration of the components in the NCs. The formation of defects is a prime characteristic of the MW synthesis of graphene materials. The implementation of MW irradiation generates an enormous amount of heat, which further creates structural defects and disorders in the graphene structure. The intensity ratio of the D and G bands (*I*_D_/*I*_G_ ratio) was calculated to evaluate the defects in the NCs. For GV, the *I*_D_/*I*_G_ ratio is found to be 0.44. However, the ratio is increased to 0.88 for GVF NC. Such a significant enhancement in the *I*_D_/*I*_G_ ratio indicates that the introduction of Fe-based components caused more disorder and defects in the carbon structure [36]. Furthermore, the peak at ≈2700 cm^−1^ represents the characteristic 2D band of graphene. The Raman pattern of rGO represents such characteristic D band at ≈1343 cm^−1^, G band at ≈1582.4 cm^−1^, and 2D band at ≈2690 cm^−1^, respectively (Figure 3d). It is interesting to note that, while GV displays a lower value, GVF displays a higher *I*_D_/*I*_G_ ratio than that of rGO (0.79). Notably, the induction of defects in graphene structures through NC formation generally leads to improved magnetic properties [37–39]. Therefore, the GVF is expected to display enhanced magnetic characteristics owing to the induction of higher defects and the presence of α-Fe_2_O_3_.

**Figure 4:**
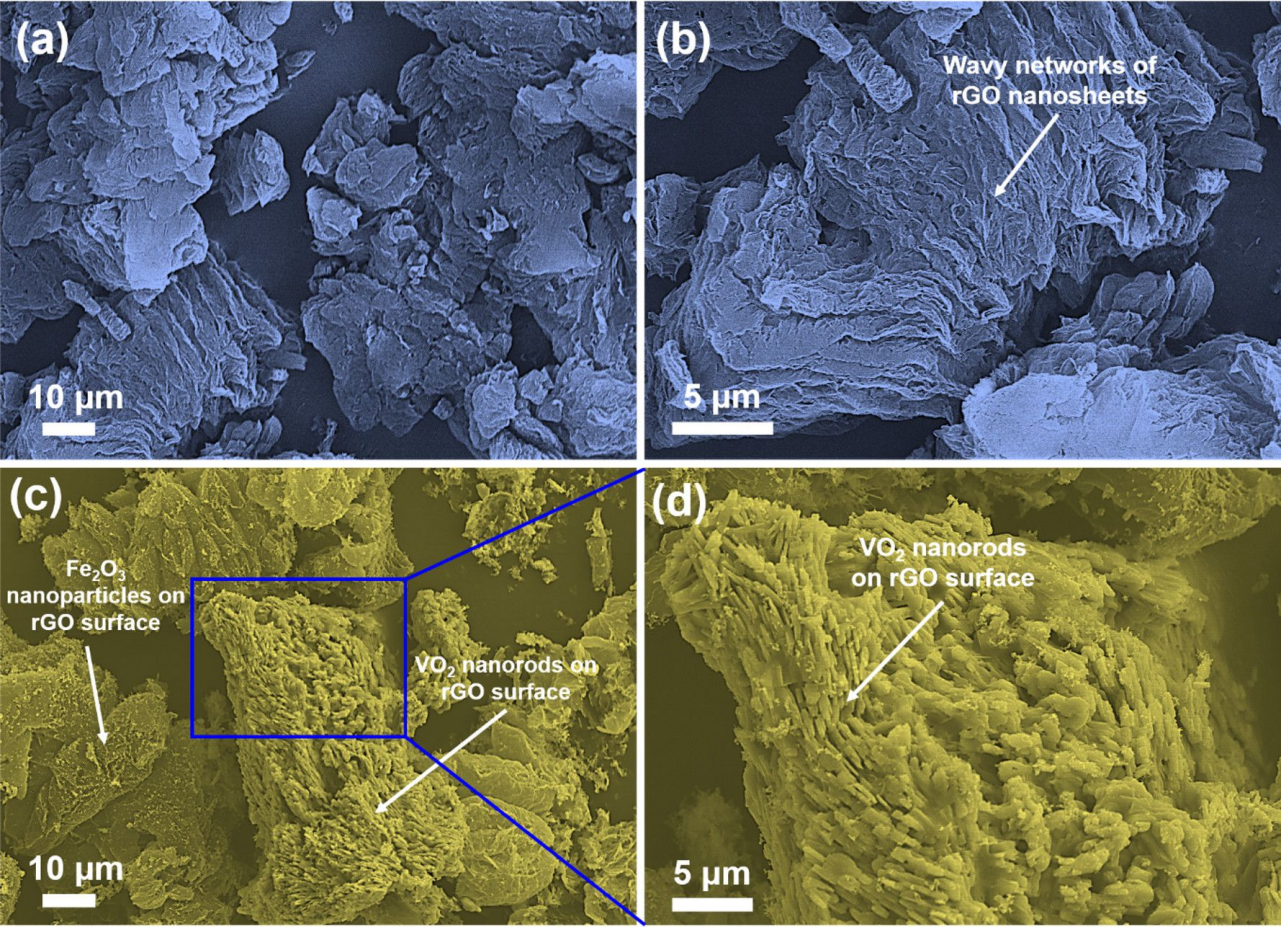
FESEM images of (a, b) rGO and (c, d) GVF NC at lower and higher magnifications.

**Figure 5:**
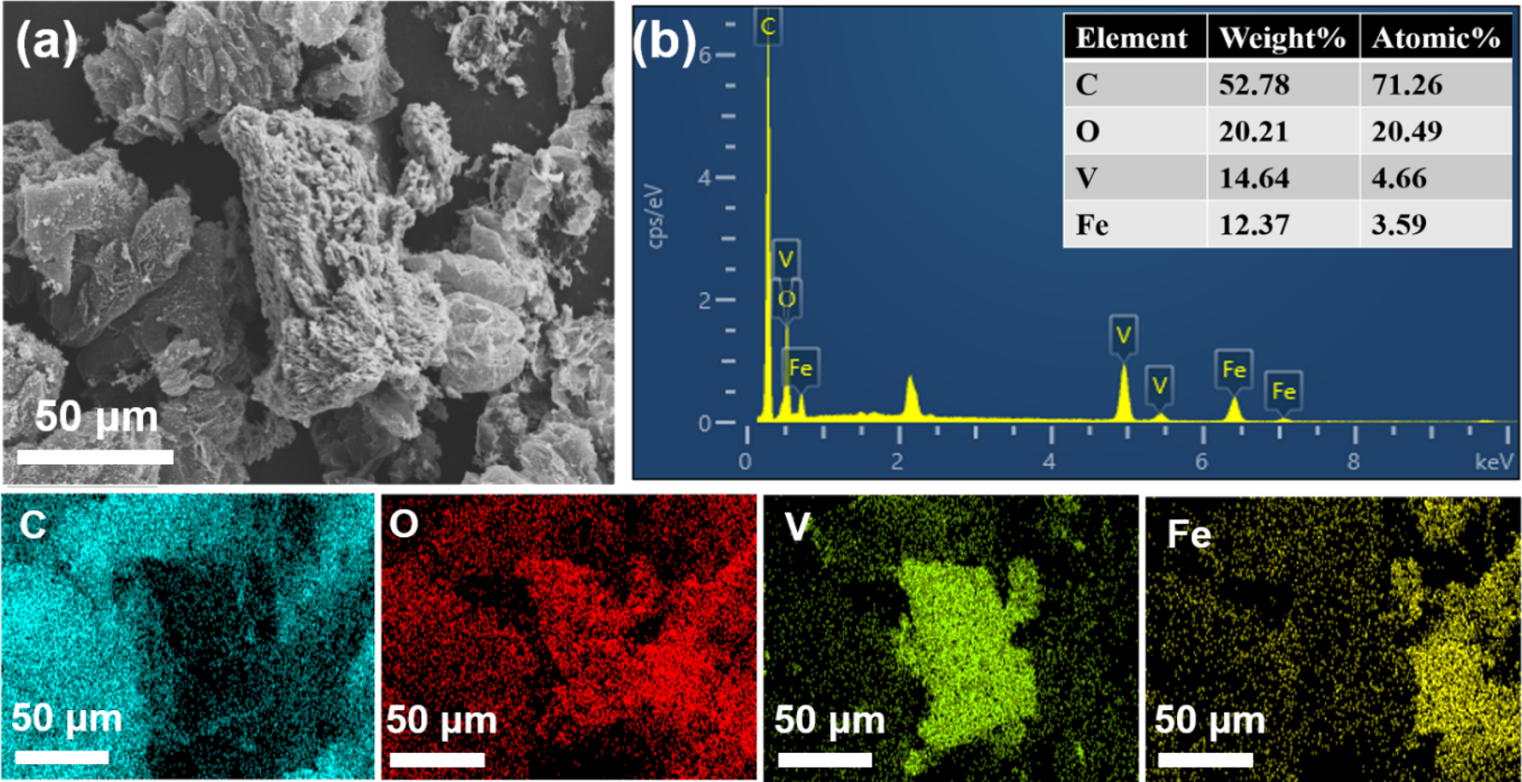
EDX analysis of GVF NC: (a) SEM image (scale bar – 50 μm), (b) EDX spectrum, and the corresponding elemental mapping showing the distribution of C, O, V, and Fe.

Figure 4a,b represents FESEM images of rGO at low and high magnifications. As shown, the morphological analyses represent the wavy-like networks of rGO nanosheets, demonstrating the exfoliation of graphene sheets. On the other hand, the SEM micrograph of GVF demonstrates the dispersion of the nanorods of VO_2_ and NPs on Fe_2_O_3_ on the graphene surface (Figure 4c,d). It is important to note that the morphological analysis of GVF displays a porous nature, which is also favorable for demonstrating improved magnetic characteristics due to alterations in the electronic structure. To further comprehend the elemental composition of GVF, the elemental analysis was also performed, and the corresponding elemental distribution and EDX spectrum are shown in Figure 5a,b. The SEM image displays a wide-range distribution of metal oxide components on the graphene surface. Furthermore, the corresponding elemental mapping demonstrates a uniform distribution of C, O, V, and Fe elements, which could be accounted for the presence of VO_2_ and iron oxide phases. Additionally, the EDX spectrum also confirms the presence of these elements.

**Figure 6:**
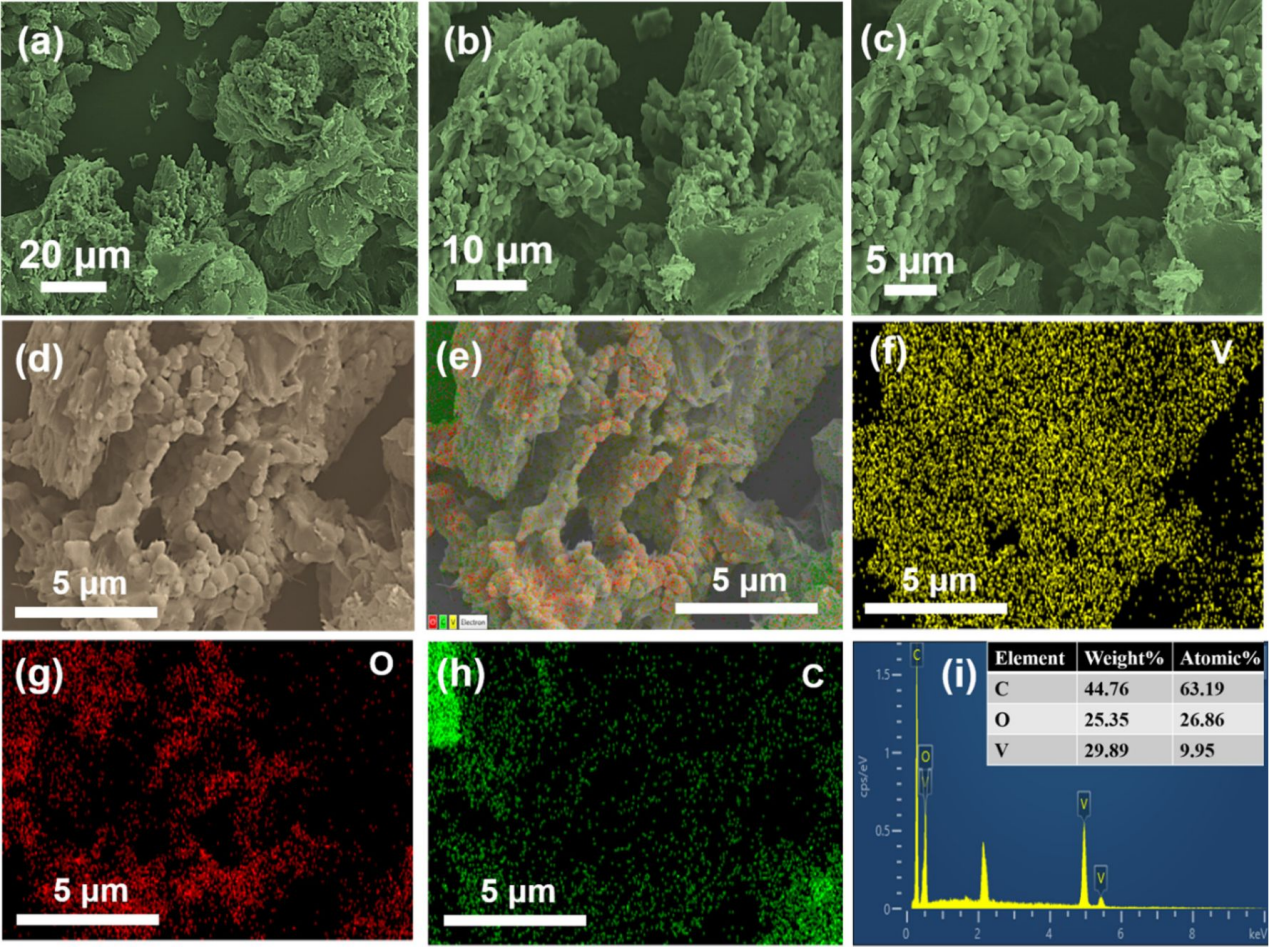
Morphology analysis of GV: (a–c) SEM images at lower and higher magnifications; (d–h) SEM image (scale bar – 5 μm) and corresponding elemental mapping displaying the uniform distribution of V, O, and C elements; (i) EDX spectrum.

For a comparative study, the morphological analysis of the GV was performed, and the corresponding FESEM images are shown in Figure 6a–c. As shown in the SEM micrographs, at various magnifications, the spherical-shaped VO_2_ particles are covered on the graphene surfaces. Notably, such spherical particles of the metal oxides are also deposited on the edges of the graphene sheets (Figure 6d). The corresponding elemental mapping further demonstrates the presence of elements such as V, O, and C in the GV (Figure 6e–h). Additionally, the EDX spectrum also confirms the presence of these elements in the NC (Figure 6i).

**Figure 7:**
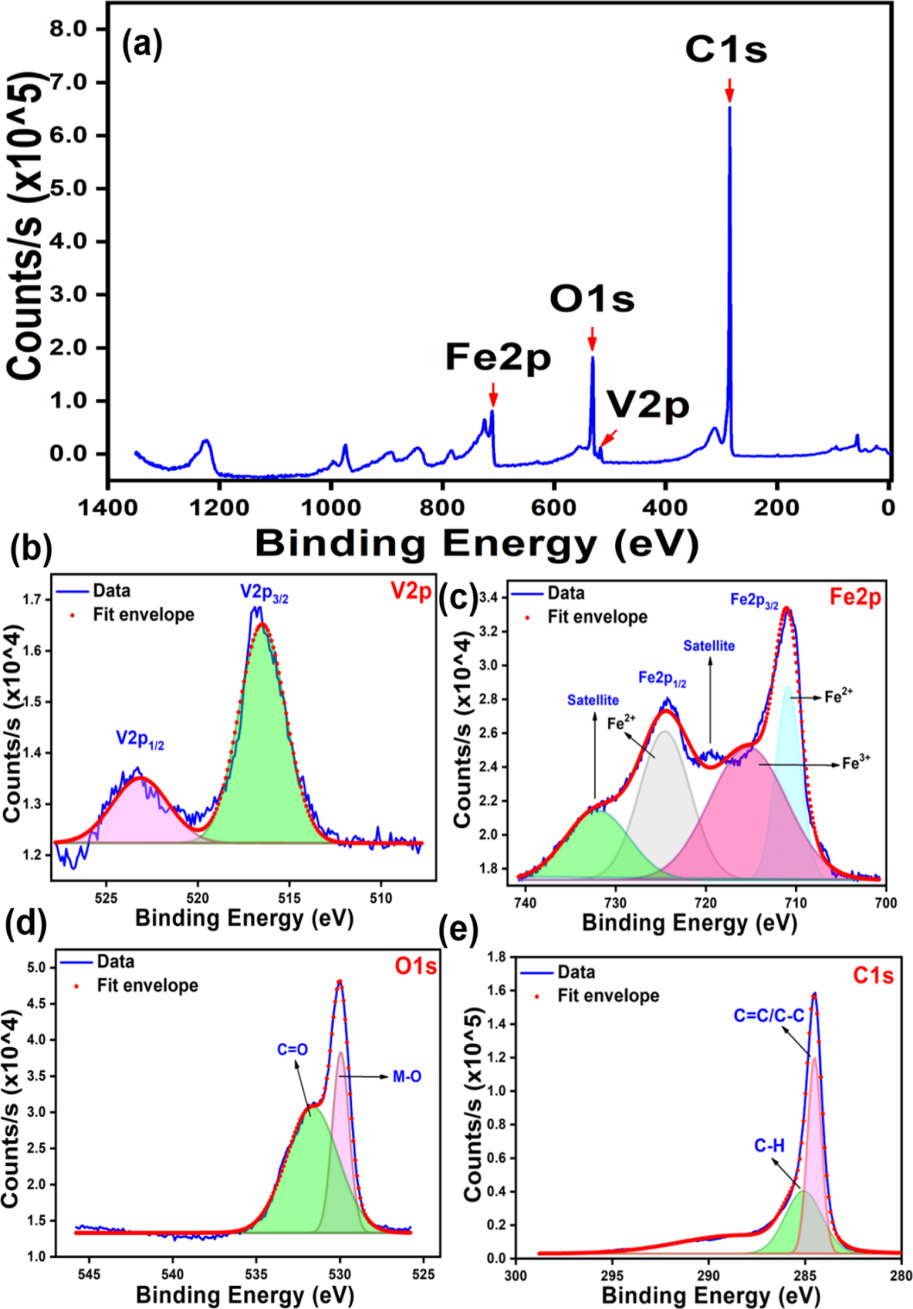
XPS analysis of GVF NC: (a) survey spectrum; high-resolution spectra of (b) V 2p, (c) Fe 2p, (d) O 1s, and (e) C 1s.

Further, to understand the surface electronic arrangement of the elements present in GVF, XPS analysis was performed. Figure 7a represents the survey spectrum, which confirms the presence of V, Fe, and O (derived from the metal oxide counterparts), along with the C element (derived from rGO). The data thus obtained was in coherence with the reported results from XRD and EDX analysis. Additionally, the high-resolution XPS spectrum of V 2p designates two major peaks at 516.5 and 523.3 eV, which can be ascribed to the V 2p_3/2_ and V 2p_1/2_, respectively (Figure 7b) [40]. On the other hand, the high-resolution XPS spectrum of Fe 2p reveals two XPS peaks centered at ≈710.8 and ≈724.5 eV, which correspond to Fe 2p_3/2_ and Fe 2p_1/2_ levels, respectively (Figure 7c). It is noteworthy to mention that the weak satellite peak at ≈719.5 eV indicates the formation of iron oxide in the form of Fe_2_O_3_, rather than its other counterparts [41]. The deconvolution of O 1s results in the formation of two major peaks at ≈530 and ≈531.6 eV (Figure 7d). While the peak at ≈530 eV can be designated to the metal–oxygen (M–O) bond, the other peak at ≈531.6 eV could be assigned to C=O of rGO. Additionally, the high-resolution XPS spectrum of C 1s represents a major peak at 284.5 eV, corresponding to the C=C/C–C bond, and a minor peak at 284 eV corresponding to the C–H bond of rGO (Figure 7e). Therefore, XPS analysis confirms the presence of distinct metal oxides and rGO in the GVF.

**Figure 8:**
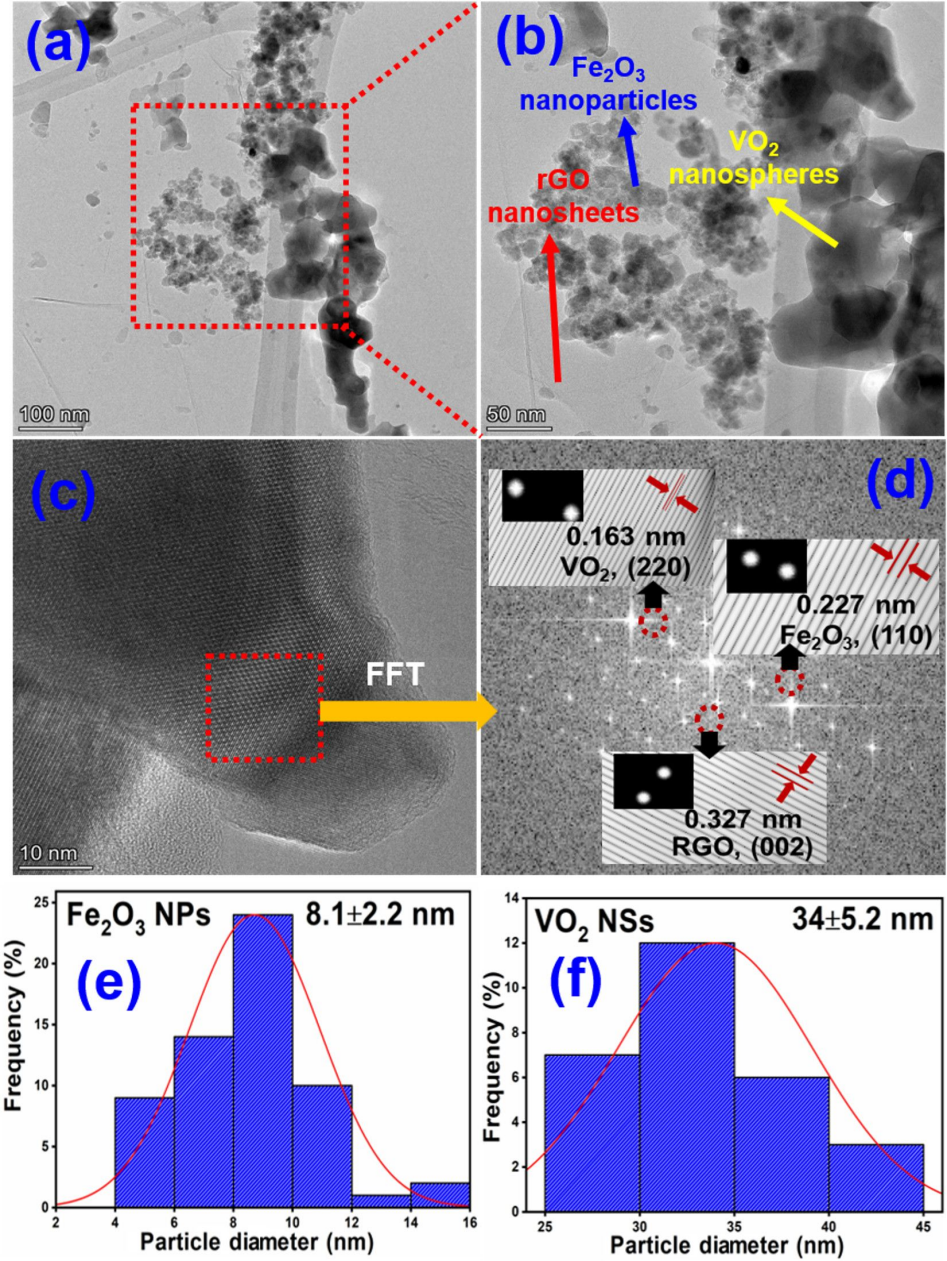
HRTEM analysis of GVF NC: (a, b) HRTEM images at low and high magnifications; (c, d) HRTEM images with corresponding FFT images; (e, f) average particle diameter of Fe_2_O_3_ NPs and VO_2_ NSs.

The detailed structure of the GVF was monitored by HRTEM analysis. The corresponding images are shown in Figure 8a–f. As shown in Figure 8a, the rGO nanosheets are found to be transparent and thin in nature. In the magnified HRTEM image, an agglomerated dispersion of Fe_2_O_3_ NPs and VO_2_ nanospheres (NSs) was visible on the thin rGO surfaces. Notably, a clear difference between the size of Fe_2_O_3_ NPs (average particle diameter – 8.1 ± 2.2 nm) and VO_2_ NSs (average particle diameter – 34 ± 5.2 nm) indicates the formation of these two different types of metal oxides on top of the rGO nanosheets (Figure 8e,f). Furthermore, the FFT analysis displays three planes with the d-spacing of 0.163, 0.227, and 0.327 nm, corresponding to the (220) plane of VO_2_, (110) plane of Fe_2_O_3_, and (002) plane of rGO, respectively (Figure 8c,d). Overall, the detailed HRTEM analysis of GVF reveals the existence of two types of metal oxide on the rGO surface, which agrees with the previous SEM, XRD, and XPS analysis.

**Figure 9:**
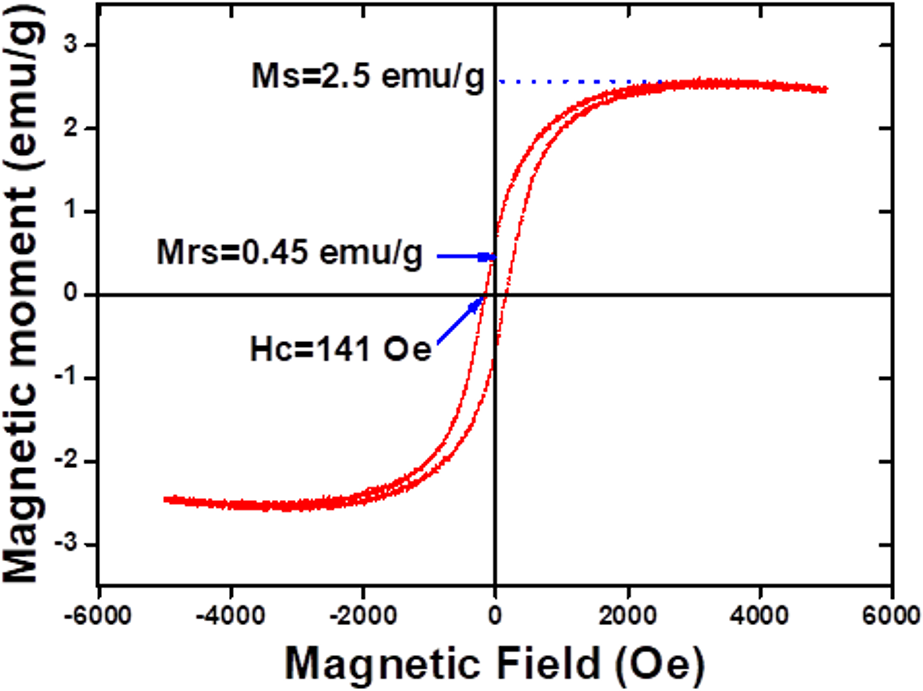
Magnetic properties of GVF NC: Room-temperature *M*–*H* curve (VSM).

The magnetic properties of the GVF investigated at room temperature under an applied magnetic field ranging from −6000 Oe to 6000 Oe is shown in Figure 9. Herein, the saturation magnetization (Ms) value for GVF is reported to be 2.5 emu/g with a magnetic retentivity (Mrs) of 0.45 emu/g and coercivity of 141 Oe. Furthermore, it is speculated that the magnetic property in the case of GVF originates mostly from α-Fe_2_O_3,_ with rGO being weakly magnetic and VO_2_ being a non-magnetic material [42–45]. The presence of magnetic behavior in GVF further promotes the occurrence of iron oxide nanoparticles. Moreover, the low magnetic moment of GVF compared to that of native α-Fe_2_O_3_ nanoparticles (mostly synthesized through the Fe-based aqueous salt) could account for the low levels of α-Fe_2_O_3_ formed during the MW-assisted synthesis process while using ferrocene as the precursor. Additionally, it is to be noted that the formation of α-Fe_2_O_3_ nanoparticles in GVF originates from the Fe in the precursor (ferrocene). To further increase the magnetic behavior of GVF, the amount of precursor should be increased, which would result in a higher concentration of α-Fe_2_O_3_ nanoparticles. These composites could be applied in many areas of biomedicine, including contrast agents for MR imaging, cancer theragnostics, and tissue engineering.

## Conclusion

In conclusion, a ternary NC consisting of α-Fe_2_O_3_, VO_2_, and rGO was successfully synthesized through a simple, efficient, and low-cost MW approach. Compared to the reported synthetic approaches, the current technique is found to be beneficial in terms of time efficiency and cost-effectiveness. The synthesized NC was characterized through various techniques, including XRD, Raman, and XPS analyses. The morphological study revealed the deposition of α-Fe_2_O_3_ NPs and VO_2_ nanorods on the rGO surface. Owing to the presence of magnetic components, the ternary NC displayed good magnetic characteristics at room temperature to showcase its potentiality in advanced biological applications. Further optimization of MW power and other reaction conditions can improve the characteristics of the composite, which could be the future prospects of the current work. Lastly, the current work opens a new door for synthesizing magnetic composites based on graphene materials along with metal oxides.

## Data Availability

Data generated and analyzed during this study is available from the corresponding author upon reasonable request.
